# Outcomes of neonates born through meconium-stained amniotic fluid pre and post 2015 NRP guideline implementation

**DOI:** 10.1371/journal.pone.0289945

**Published:** 2023-08-10

**Authors:** Ashley L. Saint-Fleur, Héctor E. Alcalá, Shanthy Sridhar

**Affiliations:** 1 Department of Pediatrics, Stony Brook Children’s Hospital, Stony Brook, New York, United States of America; 2 Department of Behavioral and Community Health, School of Public Health, University of Maryland, College Park, Maryland, United States of America; 3 Program in Oncology, University of Maryland Marlene and Stewart Greenebaum Comprehensive Cancer Center, Baltimore, Maryland, United States of America; 4 Department of Pediatrics, Division of Neonatology, Stony Brook Children’s Hospital, Stony Brook, New York, United States of America; St Paul’s Hospital Millennium Medical College, ETHIOPIA

## Abstract

A shift in the Neonatal Resuscitation Program (NRP) guidelines occurred in 2015 from routine intubation and endotracheal suctioning of all meconium-stained non-vigorous infants towards less aggressive interventions based on response to initial resuscitation. This study aims to examine the impact of this change on outcomes of non-vigorous infants born through meconium-stained amniotic fluid at a level III academic NICU encompassing years before and after the change in guideline. This single-center retrospective study compared NICU therapies and clinical outcomes of 117 non-vigorous newborns pre-guideline implementation to 106 non-vigorous newborns post-guideline implementation. Nearly two thirds of infants in the pre-guideline cohort received endotracheal suctioning with recovery of meconium compared to less than a third of infants in the post-guideline cohort (*p<0*.*01*). Though a higher proportion of the pre-guideline cohort were admitted to the NICU for respiratory issues compared to the post-guideline cohort, the two groups did not differ significantly with regard to morbidity and therapies. Despite a marked reduction in rates of intubation and endotracheal suctioning, there is no difference in outcomes between pre-guideline implementation vs post-guideline implementation in non-vigorous meconium-stained infants, supporting the recent NRP guideline change and highlighting the benefit of expectant management.

## Introduction

Meconium-stained amniotic fluid (MSAF), which occurs in 8–20% of all deliveries, remains a major perinatal clinical concern due to its association with meconium aspiration [1, 2]. It has long been observed that there is a key association between MSAF and fetal distress via several mechanisms, including airway obstruction, inflammation, and surfactant inactivation [2–5]. This can manifest as meconium aspiration syndrome (MAS), which often reflects a spectrum of disorders occurring in newborn infants born with MSAF, ranging from mild and short-term tachypnea to severe respiratory distress requiring maximum therapies and implying potential mortality [3, 5, 6].

Since airway obstruction by meconium was long believed to be an important cause of meconium aspiration syndrome, a combined obstetrical and pediatric approach to remove meconium from the infant’s airway was practiced from the mid-1970s to early 2000s [5]. Historically, Neonatal Resuscitation Program (NRP) has recommended routine intubation and endotracheal suctioning of non-vigorous infants born with MSAF to prevent perinatal aspiration of meconium [7]. This initial approach to prevention of MAS was intended to keep the infant from inhaling upon delivery the MSAF that was in the trachea, oropharynx, and nasopharynx [1]. Despite a lack of strong evidence supporting this strategy, intubation and suctioning the tracheae of all meconium-stained infants was widely adopted [8]. However, this is not without its risks as it may contribute to other morbidities, such as desaturation, bradycardia, and pneumothorax [4, 7].

As such, guidelines have continued to evolve leading to the meconium debate: to suction or not to suction. A shift in the NRP guidelines occurred in 2015 when the 7^th^ edition cited that there is insufficient evidence to recommend routine endotracheal intubation and suction in non-vigorous infants born through MSAF for the prevention of MAS [9]. The current recommendation emphasizes initiation of positive pressure ventilation (PPV) based on response to initial resuscitation during the first minute of life [9]. Routine intubation for tracheal suction in this setting is not suggested due to insufficient evidence recommending its practice (Class IIb, LOE C-LD) along with greater value being placed in harm avoidance [10].

Nonetheless, the role of neonatal suctioning of non-vigorous infants born with MSAF remains controversial, as definitive evidence does not exist to support nor to refute this practice in recent studies [5, 11, 12]. Furthermore, unlike previous guideline changes, this recommendation is not based on large randomized controlled trials raising concerns regarding its safety and efficacy [7]. This study will contribute towards filling in gaps in the literature by examining the impact of this change on outcomes of non-vigorous infants born through MSAF at a level III academic NICU encompassing years before and after the change in guideline, allowing for a better understanding of the impact of these guidelines on patient outcomes.

## Methods

### Sample

This study took place in a suburban, academic medical center. The delivery hospital has approximately 4,000 deliveries per year and admits sick infants to a level III NICU. The resuscitation team consisted of physicians and nurse practitioners who were all certified in NRP. Physicians included trainees, namely pediatric residents and neonatology fellows, as well as attending physicians. As new trainees entered their residency or fellowship training, they were certified in the current NRP guidelines and supervised during resuscitations based on their skill and level of training. Our data were extracted from medical records of mothers and non-vigorous newborns born through MSAF. Our institution adopted 7^th^ edition NRP guidelines in October 2016. We compared patients before and after this guideline change was implemented. The pre-guideline change time period encompassed two years: January 1, 2014-December 30, 2015. The post-guideline change time period was also two years: January 1, 2017-December 30, 2018. Medical records of eligible participants were collected during these time frames.

Inclusion criteria included: [1] non-vigorous infants born in the selected time period, [2] birth through MSAF of any consistency, [3] gestational age of 35–42 weeks, and [4] admission to the institution’s NICU. Infants were excluded if there were major congenital malformations/anomalies present at birth (i.e. critical congenital heart defects, trisomies, neuromuscular malformations, abdominal wall defects, CNS malformations). Of note, non-vigorous was defined as heart rate <100 beats per minute (bpm), decreased muscle tone, and/or an infant that was not breathing [7, 13]. During the “pre” time period, MSAF was noted in 411 (~10%) deliveries, yielding 117 (28%) that were non-vigorous and met the study inclusion/exclusion criteria. During the “post” time period, MSAF was noted in 452 (~11%) deliveries, yielding 106 (23%) newborns that were non-vigorous and met the study inclusion/exclusion criteria.

Ethical approval was obtained from the university institutional review board (IRB ID: IRB2020-00438). General waiver of informed consent was received. Authors had access to medical record numbers during data collection and this information was stored in a password protected shared drive only accessible to the study investigators. These identifiers were destroyed and deleted after the study was completed. The data were analyzed anonymously. The data will be protected for a minimum of seven years, as per our local IRB.

### Measures

Collected data included maternal demographics, obstetric and fetal complications, mode of delivery, and meconium consistency. Fetal distress diagnosis was based on fetal heart rate tracings (category II and III) [14]. Meconium was classified as thin (lightly stained, yellow or green) or thick (pea soup, muddy and particulate) consistency [15, 16]. Neonatal data included gestational age, birth weight, sex, APGAR scores, and reason for admission. Delivery room outcome data included positive pressure ventilation, intubation and endotracheal suction, and meconium recovery. Respiratory outcome data included respiratory distress syndrome (RDS), transient tachypnea of newborn (TTN), pneumothorax, and meconium aspiration syndrome (MAS).

TTN is a diagnosis of exclusion and cannot be made until other causes of respiratory distress have been ruled out. Clinical symptoms of tachypnea, grunting, nasal flaring, retractions as well as chest x-ray findings suggestive of retained lung fluid (i.e. fluid in the fissures, edema of the interlobar septae, streaky pulmonary interstitial infiltrates) help guide the diagnosis [17, 18]. The diagnosis of RDS was based on clinical symptoms (i.e, tachypnea, grunting, subcostal and intercostal retractions, nasal flaring, and/or cyanosis) and a chest radiograph consistent with a reticulogranular appearance to the lung fields with or without low lung volumes and air bronchograms within the first 24 hours of life [7, 19]. Pneumothorax was diagnosed if the infant had air in the pleural space diagnosed via a chest radiograph [20]. MAS was diagnosed in infants born via MSAF with poor lung compliance and hypoxemia with need for oxygen supplementation, patchy infiltrates and hyperinflation radiographically, and in infants whose symptoms could not be otherwise explained [2].

The need for oxygen, mechanical ventilation (including high frequency), surfactant, and duration of antibiotic therapy was documented. Exposure to x-ray, breastfeeding, and NICU length of stay were collected. Score for Neonatal Acute Physiology with Perinatal Extension-Version II (SNAPPE-II) was also calculated for each participant. The Score for Neonatal Acute Physiology (SNAP) developed by Richardson et al., 1993 for babies of all birth weights and validated as a predictor of mortality and morbidity, is a physiology-based score that uses 34 routinely available vital signs and laboratory test results [21–23]. Due to number and complexity of items, a second generation SNAP score (SNAP II) was validated in 1998 by Richardson et al. [24]. This score was made simpler by reducing the number of items to six and the duration for first twelve hours of admission in order to minimize the effects of early treatments. To this score were added three more perinatal variables namely birth weight, Apgar scores, and small for gestational age and was known as SNAP II with Perinatal extension (SNAPPE-II) [24–26]. The parameters included in SNAPPE-II and measured in the first 12 hours of admission to the NICU are: [1] mean blood pressure (mm Hg), [2] lowest temperature (degrees Fahrenheit), [3] PO_2_/fraction of inspired oxygen ratio as a measure of oxygenation, [4] lowest serum pH, [5] multiple seizures, [6] urine output (mL/kg/hr), [7] 5-minute Apgar score less than seven, [8] birth weight (grams), and [9] small for gestational age. Score points are assigned for each variable with additive sum ranging from zero to greater than eighty [24]. Mild scores are defined as 0–20, moderate scores as 21–40, and severe scores as greater than 41, with higher scores corresponding to greater neonatal morbidity and mortality [27].

### Data analysis

We compared various maternal and newborn characteristics and outcome variables pre and post the implementation of NPR guidelines using Stata 16. Pre and post values were compared using chi-squared test for categorical variables and t-test for continuous variables. Logistic regression and linear regression models were used to determine the outcomes were associated with implementation of NPR guidelines, after controlling for potential confounders. All models control for chorioamnionitis, thick meconium, prolonged rupture of membranes, fetal distress, and gestational diabetes. The results are presented as adjusted odds ratios (AOR) or adjusted beta coefficients with 95% confidence intervals (CIs). Statistical significance is set at a probability value of <0.05.

## Results

### Descriptive analyses

As shown in Table 1, maternal characteristics between the pre-guideline change and post-guideline change cohorts were similar. There was no statistically significant difference in the modes of delivery, incidences of hypertensive disorders of pregnancy, gestational diabetes, chorioamnionitis, prolonged rupture of membranes, thick meconium, or fetal distress.

**Table 1:** Maternal characteristics.

	Pre-Guideline Change (n = 117)	Post-Guideline change (n = 106)	*p*
	Mean±SD	Mean±SD	
**Maternal Age (years)**	29.2±5.8	30.3±5.2	0.17
	**N (%)**	**N (%)**	
**Maternal Race**			0.86
White	86 (73.5%)	79 (74.5%)	
Non-White	31 (26.5%)	27 (25.5%)	
**Maternal Ethnicity**			0.29
Hispanic	28 (23.9%)	32 (30.2%)	
Non-Hispanic	89 (76.1%)	74 (69.8%)	
**Hypertensive Disorders of Pregnancy**	4 (3.4%)	2 (1.9%)	0.48
**Gestational Diabetes**	7 (6.0%)	11 (10.4%)	0.23
**Chorioamnionitis**	67 (57.8%)	59 (55.7%)	0.75
**Prolonged Rupture of Membranes**	23 (19.8%)	18 (17.0%)	0.59
**Thick Meconium**	70 (40.2%)	57 (53.8%)	0.36
**Mode of Delivery**			0.12
Vaginal Delivery	75 (64.1%)	57 (53.8%)	
Caesarean Delivery	42 (35.9%)	49 (46.2%)	
**Fetal Distress**	35 (30.0%)	41 (36.7%)	0.17

**P<0.05 -statistically significant

Newborn characteristics such as gestational age, birth weight, sex and presence of thick meconium were similar between the pre-guideline change and post-guideline change cohorts (Table 2). The mean gestational age was approximately 40 weeks and mean birth weight approximately 3400g. A greater proportion of the pre-guideline change cohort received endotracheal suctioning when compared to the post-guideline change cohort (61.5% vs 14.2%). This difference, along with meconium recovery, was statistically significant. Importantly, meconium recovery was only done among newborns who received endotracheal suctioning, representing a subsample of those receiving endotracheal suctioning. There was no statistically significant change in performance of positive pressure ventilation.

**Table 2:** Newborn characteristics.

	Pre-Guideline Change (n = 117)	Post-Guideline Change (n = 106)	*p*
	Mean±SD	Mean±SD	
**Gestational Age** *(weeks)*	40.0 ± 1.3	39.6 ± 1.3	0.43
**Birth weight** *(grams)*	3464 ± 490	3419 ± 498	0.50
**APGAR score**			
1 minute	4.34±2.0	3.99±0.2	0.22
5 minutes	7.40±1.5	7.14±1.9	0.26
	**N (%)**	**N (%)**	
**Male gender**	68 (58.1%)	62 (58.5%)	0.96
**Positive Pressure Ventilation (PPV)**	88 (75.2%)	73 (68.9%)	0.29
**Endotracheal suctioning**	72 (61.5%)	15 (14.2%)	**<0.01** **
Meconium recovered	30 (25.6%)	3 (2.8%)	**<0.01** **
**Thick meconium**			0.362
**No**	47(40.17%)	49 (46.23%)	
**Yes**	70 (59.83%)	57 (53.77%)	

**p<0.05 statistically significant

### Regression analyses

After adjusting for APGAR score at 1 minute, APGAR score at 5 minutes, chorioamnionitis, thick meconium, prolonged rupture of membranes, and gestational diabetes, the two groups did not differ significantly with regard to incidence of any of the outcomes, including incidence of meconium aspiration syndrome, pneumothorax, RDS, TTN, or need for supplemental oxygen/ventilatory support and exposure to x rays (Table 3). Though a higher proportion of the pre-guideline cohort were admitted to the NICU for respiratory issues compared to the post-guideline group (64.1% vs 59.4% respectively), this difference was not significant in logistic regression analyses that accounted for potential confounders. The average SNAPPE-II scores corresponded to the mild illness severity for both groups.

**Table 3:** Newborn therapy & outcomes.

	Pre-Guideline Change (n = 117) N (%)	Post-Guideline Change (n = 106) N (%)	Adjusted Odds Ratio or Beta Coefficient ^a^ (95% Confidence Interval)
**NICU Respiratory Admission**	75 (64.1)	63 (59.4)	1.33 (0.69, 2.57)
**Meconium Aspiration Syndrome**	27 (23.1)	18 (17.0)	0.63 (0.31–1.28)
**Respiratory Distress Syndrome**	55 (47)	43 (40.6)	1.47 (0.56, 3.83)
**Transient Tachypnea of Newborn**	34 (29.1)	41 (38.7)	0.86 (0.48, 1.53)
**Pneumothorax**	15 (12.8)	15 (14.2)	0.56 (0.25, 1.26)
**Exposure to X-rays**	90 (76.9)	80 (75.5)	0.84 (0.42, 1.71)
**Need for Supplemental Oxygen**	70 (59.8)	70 (66%)	0.88 (0.51, 1.54)
**Need for Ventilatory Support**	22 (18.8)	19 (17.9)	1.21 (0.54, 2.75)
**Duration of Antibiotics** (*days*) ^b^	3.7 ± 4.1	1.75 ± 1.5	0.19 (-0.63, 1.01)
**Surfactant Therapy** ^c^	1 (0.9)	5 (4.7)	-
**Length of Stay** (*days*) ^b^	7.6 ± 7.4	7.7 ± 7.6	-0.01 (-1.74, 1.72)
**Breastfeeding**	75 (64.1)	76 (71.7)	0.87 (0.48, 1.53)
**SNAPPE-II ^b^** ^d^	18.0 ± 14.7	19.0 ± 14.2	-1.58 (-4.68, 1.52)

^a^ Dichotomous outcomes are modeled with logistic regression models and continuous outcomes are modeled with linear regression models

^b^ Data are given as mean ± SD

^c^ Unable to calculate OR with low overall incidence of the outcome

^d^ Score for Neonatal Acute Physiology with Perinatal Extension-II

## Discussion

Results indicated that nearly two thirds of infants in the pre-guideline change cohort received endotracheal suctioning with recovery of meconium compared to less than a third of infants in the post-guideline change cohort, demonstrating a statistically significant change in practice. In addition, though a slightly higher proportion of the pre-guideline cohort were admitted to the NICU for respiratory issues compared to the post-guideline group (64.1% vs 59.4%), this difference was not statistically significant in logistic regression analyses. Furthermore, the two groups did not differ significantly with regard to incidence of respiratory outcomes, including RDS, MAS, TTN, need for supplemental oxygen/ventilatory support, or exposure to x-rays, or with regard to duration of antibiotics, breastfeeding, or length of stay. SNAPPE-II scores were also comparable, suggesting intubation and endotracheal suctioning did not have a significant impact. Moreover, the two groups had comparable maternal and infant characteristics, ensuring that the effect of the guideline change was not confounded by other known factors.

The results of this study were consistent with previous studies comparing outcomes pre- and post-guideline implementation reporting a nonsignificant difference in the rate of MAS or other measures of neonatal morbidity [5, 8, 28–30]. Similar to our findings, Myers *et al*. [31] reported that delivery room intubations of term infants with MSAF had a statistically significant decline while admission to NICU, need for respiratory support, and length of stay remained unchanged. Additionally, in their systemic review and meta-analysis, Phattraprayoon *et al*. [13] revealed no statistically significant difference in MAS, need for respiratory support, pneumothorax, blood-culture positive sepsis, or length of stay, supporting the practices in the NRP 2015 guideline. In contrast, Chiruvolu *et al*. [7], revealed an increase in NICU admission for respiratory problems, mechanical ventilation, oxygen use, and surfactant therapy in meconium-stained non-vigorous infants. However, this study was limited by presence of confounding variables.

It is possible that the failure of endotracheal suctioning to prevent MAS could be attributed to occurrence of aspiration of meconium in utero and inability to retrieve meconium from trachea due to migration to distal airways [29]. In addition, other pathophysiological mechanisms of MAS, including surfactant inactivation, persistent pulmonary hypertension, and inactivation of Toll-like receptors may also contribute to the lack of a benefit seen with suctioning [2, 3, 5, 29, 32]. The majority of cases of severe MAS requiring mechanical ventilation have a background of non-reassuring fetal heart rate monitoring, neonatal depression, and in many cases thick meconium, which could support the theory of in utero damage [4, 5]. Furthermore, studies reporting a lack of correlation between presence of meconium in trachea and clinical severity of MAS also raise doubts on the utility of performing endotracheal suctioning [33].

## Conclusions

Our results suggest there is no difference in outcomes between pre-guideline implementation vs post-guideline implementation in non-vigorous meconium-stained infants. Our institution embraced the recommendations suggested by the NRP’s seventh edition and markedly reduced rates of intubation and suctioning in the delivery room without any associated increase in morbidity. These findings support the recent NRP guideline change and highlight the benefit of expectant management. Nonetheless, there are study limitations that exist. The single center retrospective study design limits our generalizability and absence of data on potential confounders, such as level of skills of the delivery attendants. Furthermore, we could not control for provider to attention to implementation of the new guidelines, but all providers are undergo routine NRP recertifications to maintain skill set and ensure continued adherence to the guidelines through monthly task force meetings held in the NICU. In addition, some physicians might be reluctant to diagnose a respiratory disorder as MAS unless there are classic chest radiographical findings, which can lead to an underdiagnosis [5]. Unfortunately, chest radiographical patterns in MAS are diverse, and range from diffuse patchy infiltrates or consolidation to a hypovascular (or even an apparently normal) appearance [5, 34]. As stated by Katz and Bowes [35] “the severity of X-ray findings may not always correlate with the clinical disease.” Future studies evaluating implications of this guideline change may highlight the impact of the decrease in neonatal intubation on procedural experience and skill of practitioners, particularly trainees, and evaluate the need to provide additional learning opportunities to all levels of experience.

## Supporting information

S1 Data (XLSX)
